# Critical monitoring and control factors for achieving food defense criteria

**DOI:** 10.1111/risa.70025

**Published:** 2025-04-04

**Authors:** Elaine Andrade, Flavio Andrade, Gilberto Oliveira, Otniel Freitas‐Silva

**Affiliations:** ^1^ Universidade Federal do Estado do Rio de Janeiro / Programa de Pós Graduação em Alimentos e Nutrição (UNIRIO/PPGAN) Rio de Janeiro Brazil; ^2^ Universidade Federal Fluminense / Programa de Pós Graduação em Engenharia de Produção (UFF/PPGEP) Niterói Brazil; ^3^ Universidade Federal do Rio de Janeiro /Instituto de Relações Internacionais e Defesa (UFRJ/IRID) Rio de Janeiro Brazil; ^4^ EMBRAPA Agroindústria de Alimentos Rio de Janeiro Brazil

**Keywords:** critical success factors, food defense, food fraud, food producers, food safety, intentional contamination

## Abstract

Food can be contaminated by physical, chemical, biological, and radiological hazards. Industry and regulatory agencies have developed the Food Safety Management System, based on the principles of Hazard Analysis and Critical Control Points, to be effective against unintentional food safety risks. However, there is the intentional contamination of food to cause harm to consumers, customers, or companies, and prevention requires a different approach to controlling unintentional food safety risks; this concept is known as food defense. There are certification standards that include food defense requirements referenced to the Global Food Safety Initiative (GFSI), such as the British Retail Consortium, International Featured Standards, Food Safety System Certification 22000, and Safe Quality Food Institute. In the context of food producers, it is crucial to understand the critical success factors for implementing standards involved in food defense and increasing the effectiveness and sustainability of exporting agroindustries. The present study aimed to map the essential success factors for implementing food defense in Brazilian companies already certified by the standards recognized by the GFSI, to take advantage of this expertise for companies seeking to implement food defense. Quantitative and qualitative research was conducted by a survey of the perception of those responsible for implementing food safety standards regarding the relevance of food defense requirements to guarantee food protection. Data analysis was performed through correspondence analysis. It was possible to identify groups and formulate a reduced list of priority criteria for the implementation of food defense, to facilitate and accelerate the adaptation process of companies not yet certified, helping to raise the level of food safety for consumers, in addition to contributing to economic growth through new entrants in the export and import chain.

## 1 INTRODUCTION

Food defense in the European food industry is a relatively new concept in many EU countries, unlike in the United States, where this concept originated. It was officially defined by the Public Health Security and Bioterrorism Preparedness and Response Act of 2002 (Bioterrorism Act, 2002), adopted after the terrorist attacks of September 11, 2001. The main purpose of the Bioterrorism Act is to protect the food supply from intentional contamination (Bogadi et al., 2016).

The Global Food Safety Initiative (GFSI) definition of food defense is the process of ensuring the safety of food, ingredients, feed, or packaging, from all forms of intentional malicious attack, including ideologically motivated attacks leading to contamination or unsafety of products (GFSI Benchmarking Requirements Version 2020, 2020).

GFSI is a global initiative that aims to improve food safety by harmonizing and implementing best practices in food safety systems worldwide. This initiative aims to ensure consumer confidence in food products by promoting food safety standards that protect both consumers and companies in the supply chain (GFSI Benchmarking Requirements Version 2020, 2020). In addition, the FSMA IA (Food Safety Modernization Act—Intentional Adulteration) is a part of the American legislation, focused on preventing intentional adulteration of food, as part of the efforts to modernize food safety in the United States. FSMA IA requires food companies to implement food defense measures to prevent deliberate attacks, including acts of bioterrorism (FDA, 2016).

PAS96, one of the main food defense standards, focuses on mitigating the risks related to intentional food adulteration and sabotage of the food chain. Unlike other food safety management systems, such as those of GFSI and FSMA IA, PAS96 covers a broader approach, including the scope of food fraud or EMA (Economic Motive Attacks), that is acts of fraud that are economically motivated. This inclusion of food fraud in PAS96 is distinct from the GFSI and FSMA IA standards, which mostly focus on food defense against ideological or terrorist attacks, with GFSI including food fraud under the concept of VACCP (Vulnerability Assessment Critical Control Points), while FSMA IA focuses on risk mitigation measures linked to intentional and terrorist attacks, but not necessarily economic fraud (GFSI Benchmarking Requirements, 2020; Food Safety Modernization Act, 2011).

It is important to note that compliance with PAS96 does not equate to compliance with GFSI or FSMA IA. PAS96 takes a distinct approach to food fraud, which is not fully addressed by the other two standards. Therefore, companies implementing PAS96 should be aware that compliance with this standard does not guarantee compliance with the specific requirements of GFSI or FSMA IA (The British Standards Institution, 2017).

In PAS96, the concept of TACCP (Threat Assessment Critical Control Points) is used to assess and mitigate the risks of intentional attacks on food safety, with a focus on identifying critical points where such attacks may occur. This is different from the VACCP concept used in GFSI, which, although it also assesses vulnerabilities, has a greater focus on food fraud and risks related to fraudulent economic gains. TACCP therefore includes a broader range of risks, such as the possibility of fraud or sabotage, while VACCP focuses more specifically on aspects related to economic fraud (GFSI Benchmarking Requirements Version 2020, 2020; The British Standards Institution, 2017).Furthermore, using PAS96 as a reference for implementing food defense in food industries brings several advantages:

1. Comprehensive Risk Coverage: PAS96 allows for a detailed analysis of a wider range of threats, including not only the risks of terrorist and ideological attacks, but also economically motivated food fraud (EMA). This expanded aspect is a significant advantage over the GFSI and FSMA IA standards, which do not address food fraud as comprehensively (The British Standards Institution, 2017).
2. Proactive and Preventive Risk Approach: PAS96's TACCP methodology allows for a proactive and preventive approach to food defense, helping companies identify critical points in the supply chain where an intentional attack could occur. This approach makes it easier to implement corrective measures before any incident occurs, which is a significant advantage over other food defense systems that may be more reactive (The British Standards Institution, 2017).
3. Flexibility for Different Sectors: PAS96 is flexible and can be adapted to different types of food industries, regardless of the size or complexity of the supply chain. This versatility allows both large corporations and small businesses to implement an efficient food defense system, tailored to their specific needs and risks (Buchanan & Appel, 2010).
4. Improved Regulatory Compliance: For companies seeking to expand internationally, adopting PAS96 can improve compliance with the food safety requirements of different countries and increase consumer and customer confidence. This is especially relevant in markets where food defense is a requirement for the export of food products (Praia & Henriques, 2021).

Industry and regulatory bodies have developed Food Safety Management Systems based on the principles of Hazard Analysis and Critical Control Points (HACCP), which have proven effective against unintentional food safety risks. However, HACCP principles are not used to detect or mitigate intentional attacks and are therefore not relevant to the scope of food defense.

The motivation or root cause of food defense is the intention to cause harm to consumers, customers, or businesses. This approach is not the same as the motivation for food fraud, which involves economic gain. Therefore, preventing food defense requires a different approach than controlling unintentional food safety risks (HACCP) and preventing food fraud (Food Safety System Certification 22000, 2019).

However, according to the World Health Organization (2007), the food chain has undergone considerable changes in the last 50 years, becoming highly sophisticated. Although food safety has improved dramatically overall, progress is uneven and outbreaks of contamination by microbes, chemicals, and food‐borne toxins are common in many countries. Trade in contaminated food between countries increases the potential for outbreaks of foodborne illness to spread. Furthermore, the emergence of new foodborne diseases is becoming a considerable concern (Forsythe, 2013).

Risks to food safety place a greater burden on developing countries, which have limited capacity both to produce safer food and to deal with foodborne illnesses. Aid to strengthen food safety in developing countries can improve those countries' access to the global market and increase income from trade. The initiatives can also have positive repercussions on the national food supply chain, benefiting consumers and producers in developing countries (Narrod et al., 2021).

Achieving food safety poses a significant challenge due to the openness of food production, manufacturing, and marketing systems; the global nature of the food industry; the large size of production batches; the difficulties in distinguishing unintentional and intentional contamination; the ability to use food as a transmission vehicle without the need to weaponize the agent; and the wide range of microbiological, chemical, and radiological agents that can be introduced into food (Buchanan & Appel, 2010).

The World Health Organization (WHO) has identified food defense as one of the major public health threats of the 21st century since food can be an instrument for terrorist acts (World Health Organization, 2007). Intentional contamination with toxic materials can cause poisoning, death, supply chain sabotage, and, consequently, food shortages. The consequences of such acts can disrupt food exports, reduce tourism, impair economic performance, and cause political and social destabilization and public health problems. Therefore, food defense must have a multidisciplinary approach involving national and international institutions (US Department of Agriculture, 2023).

Currently, food companies are increasingly required to comply with food quality and safety standards to expand their businesses nationally and internationally (Bogadi et al., 2016).

The growing global demand for Brazilian food products imposed a need to meet increasingly stringent requirements, including specific food defense certifications. In the context of food exports, Brazilian companies need to obtain the necessary food defense certifications in compliance with international requirements. These certifications are essential to assuring importing countries that companies adopt effective protection measures against deliberate threats to food (Praia & Henriques, 2021).

The food safety system can be implemented and certified by various standards recognized by the GFSI, such as those of the British Retail Consortium (BRC), International Featured Standards (IFS), Food Safety System Certification (FSSC 22000), and Safe Quality Food Institute, which have been changing to encompass the food defense concept (Severino & Almeida, 2016).

The Intentional Adulteration Rule (Mitigation Strategies to Protect Food Against Intentional Adulteration (21 CFR part 121)), described by the Food and Drug Administration (FDA), specifies the required contents of the Food Defense Plan (FDP). An FDP is a set of written documents based upon food defense principles incorporated with a vulnerability assessment, including mitigation strategies, delineating food defense monitoring, corrective action, and verification procedures to be followed (FDA, 2019; Office of the Federal Register, 2013)).

Implementing a successful FDP requires considering several critical success factors such as the identification and assessment of risks and implementation of preventive controls, along with employee training and continuous review of the plan are some of the factors. Furthermore, collaboration with regulatory bodies, the participation of the entire supply chain, and the engagement of senior management are crucial elements for successful implementation (Developing a Food Defense Plan: A Guide, 2008).

The FDP is a set of actions to prevent malicious attacks, including threat identification, risk assessment, and determination of control measures (Manning & Soon, 2016). Preventive measures include external barriers, such as doors and fences, which must have an automatic locking system to prevent access by unauthorized individuals. These barrier types keep potential attackers away from the production company and are quite effective against intruders, but do not provide control against employees (Motarjemi & Lelieveld, 2014). When controlling access to employees, mitigation measures must be established, such as identification at the entrance to the food company and, when possible, the use of appropriate uniforms differentiated by the work sector, facilitating the recognition of unauthorized people within the establishment. Personal identification numbers or microchips should be assigned to employees, temporary workers, and service providers. Furthermore, visitors must be properly identified within the facilities and whenever possible must be accompanied by someone to guide them during the visit. Adequate training of all employees in food defense, emphasizing information necessary for the correct performance of their respective functions in a particular area, is also an important measure (Severino & Almeida, 2016).

Other measures that can be adopted include: ensuring that only authorized suppliers deliver raw materials; inspecting the packaging to verify its integrity; monitoring the entry and exit of vehicles at scheduled times and access points; recording all raw materials as well as all final products to ensure traceability and facilitate collection and withdrawal operations; formulating handling instructions and keeping an inventory of all materials identified as potentially dangerous; and maintaining a traceability system to allow products to be recalled from the market quickly and effectively in the event of an incident or detection of adulteration (Lorenzen et al., 2009).

Each organization is different and not all mitigation measures are applicable, practical, or effective for all types and sizes of companies. Therefore, the measures to be implemented will best suit the structure and meet organizational needs (Severino & Almeida, 2016). Food facilities that are significantly vulnerable to intentional adulteration are required to develop and implement an FDP based on information provided about the facility, process, product description, vulnerability assessment, mitigation strategies, food defense monitoring procedures, food defense corrective action, and defense verification procedures, including attack simulation and attempted threats to food safety (FDA, 2019).

In addition to all these precautions, when our food is grown, processed, prepared, sold, and served by others, we rely on each person in the supply chain to make the right decisions to keep our food safe. These decisions are highly impacted by the cultures of each organization along the chain, and how dimensions within these cultures enable or hinder food safety decisions and practices. GFSI defines a food safety culture as: “shared values, beliefs, and precepts that affect the mindset and behavior toward food safety throughout an organization.” However, the GFSI believes that to be successful and sustainable within a company's culture, food safety must go beyond formal regulations. Culture exists apart from written laws and regulations (GFSI, 2018).

Although the GFSI has the aim of strengthening and harmonizing food safety systems through benchmarking and working to promote mutual acceptance of certification programs in all sectors, there is still no clear standardization among the requirements recognized by the GFSI regarding the implementation of a food defense management system (Weber, 2024).

The present article aims to contribute to the standardization and prioritization of requirements, based on the analysis of different standards and the expertise of certified Brazilian companies, to facilitate the adjustment process of already certified companies and the implementation of food defense requirements by companies seeking food safety certification. Today Brazil is the largest global exporter of sugar, coffee, orange juice, soybeans, beef, and chicken, the third largest of corn, and the fourth of pork. It is also the world's largest producer of soybeans, coffee, orange juice, and sugar, the second of beef, and the third of chicken and corn. Overall, Brazil is the fourth largest exporter of agricultural products worldwide, behind only the European Union, the USA, and China (CNA, 2021).

## 2 METHODOLOGY

To identify the critical success factors of food defense in food industries, quantitative and qualitative approach research was carried out, through a survey of the perceptions (Creswell, 2007) of those responsible for implementing food safety standards regarding the relevance of food defense requirements to guarantee food protection. The survey involved quantifiable data from a population collection to describe or identify covariation between variables that can indicate causal relationships or predictive patterns of influence (Sapsford, 2007). The survey method was applied through a self‐administered questionnaire on the Internet, with a link to the electronic address sent to people responsible for the quality management or production area of food companies. The collected data were submitted to the correspondence analysis (CA).

### 2.1 Survey

The questionnaire was divided into three parts. The first one involved company characterization, where data were collected regarding the location, size, sector of activity, type of market, and company certifications. In the second part, data were obtained on food defense requirements based on 30 statements to be evaluated by experts regarding the relevance of each requirement.

The food defense attributes used in the questionnaire, described in Table 1, were selected from the list of critical controls referenced in PAS 96:2017, the UK National Standards Body's current guidance on protecting and defending food and drink from deliberate attack, developed by the British Standards Institution (BSI). BSI offers food safety certifications, such as BRCGS (Brand Reputation Compliance Global Standards) and ISO 22000, being recognized by the GFSI. In this work, PAS 96:2017 was chosen as the guide, as it provides practical guidance on how to avoid and mitigate threats to food and the food supply and is the only one that presents a risk management methodology, TACCP closely aligned with HACCP.

**TABLE 1:** Food defense attributes used in the questionnaire, selected and adapted from the list of critical controls referenced in PAS 96:2017.

Attribute number	Requirement
1	The existence of perimeter fencing throughout the factory
2	Finished product storage in a specific location
3	Identification and registration of visitors, and monitoring them throughout the visit
4	Investigation of missed deliveries
5	Control of hazardous materials
6	Control of access to key stock materials
7	Limitations on access to network services
8	System for tracking transport vehicles
9	Monitoring of vehicle access points
10	Qualification of suppliers
11	The existence of a perimeter alarm system
12	Providing employees with a list of emergency contacts
13	Physicochemical and microbiological control of raw materials
14	Proof of identity with criminal record search before hiring new employees
15	Restricted access and monitoring of third‐party access
16	Routine cyber training (security principles)
17	Finished product (retail) packaging with effective seals
18	Control of packaging labels
19	Restricted and controlled access of employees to relevant areas
20	Uniforms differentiated by sector
21	Restriction on the use of cameras and other portable electronic devices
22	CCTV monitoring/recording of perimeter vulnerabilities
23	Controlled access to the utilities area (ventilation, air conditioning, water storage, steam system, electrical system, etc.)
24	Parking of vehicles of employees and visitors outside the industrial sector
25	Access control (entrances and exits) by CHIP and PIN
26	Computer accounts closed or suspended during the dismissal process
27	Access cards and keys collected during the dismissal process
28	Differentiated recruitment for sensitive functions and/or critical roles (concerning risks)
29	Employee awareness of food safety and security
30	CCTV monitoring/recording of vulnerable areas

*Source*: Authors.

According to the attributes referenced in PAS 96:2017, each statement had five response options graded on a Likert scale of: “1—Not at all relevant,” “2—Slightly relevant,” “3—Indifferent,” “4—Relevant,” and “5—Totally relevant” (Money et al., 2005). The instrument was calibrated by six experts on the subject. The third part referred to the FDP regarding the existence of a plan and its effect on food safety culture.

Likert scales are the most frequently used variation of summation rating scales. They involve statements that express favorable or unfavorable attitudes toward the topic of interest. The respondent is asked to agree or disagree with a statement. Each response receives a numerical rating that reflects favor or disfavor. The numbers are then added to measure the respondent's attitudes (Cooper and Schindler, 2003).

To develop the online questionnaire sent to research participants, we chose the Google Forms tool, since it has several advantages, such as no cost; possibility of access at any place and time; agility in data collection and analysis of results; and ease of use, among other benefits. It can be sent to potential respondents via email or a link. Another advantage is the organization and depiction of data in graphs and spreadsheets, providing quantitative results in a more practical and organized way, and facilitating data analysis (Mota, 2019).

To increase engagement in the research and seek greater reliability in the responses, the questionnaire was answered anonymously. The complete form can be found in the Supporting Information or through the link: https://forms.gle/VCAjKJtXuAtwHNwY8.

### 2.2 Sampling

The sample was nonprobabilistic (Siegel, 2006), and selected due to the degree of specialization in food defense. The sample used was composed of representatives of food production companies with active certifications in Brazil, extracted from a public consultation database of certification standards recognized by the GFSI, which include food defense requirements, namely: Global Food Safety Standard (BRCGS) and FSSC 22000. Although the IFS Food standard is recognized by the GFSI and is used by Brazilian companies, it was not included in this study since it was not on the list of certified companies available for public consultation.

The sample was comprised of representatives of 175 companies extracted from the public consultation database available at the website: https://directory.brcgs.com/, accessed in October 2022, applying the country filter “Brazil” and the standard filter: “Food,” which contained information such as company name, category, certification body, date of issuance and expiration of certification, email of technical and commercial contacts and company telephone number, or extracted from another public consultation database available on the website: https://www.fssc.com/public‐register/, accessed in October 2022, using the command “Export all” and applying the filters: Country “Brazil,” Status: “valid,” removing the “production” option from the FCC filter of (Bio) chemicals, “production of food packaging,” “production of feed,” “provision of transport,” and the filter “statement” packaging options, animal feed, additives, and food ingredients, leaving 445 of a maximum of 33,488 potential records. The database extracted from FSSC 22000 only contained the company name and address, with no telephone numbers or contact emails.

The questionnaire was first sent in October 2022 via email to 160 technical persons of the 175 existing in the BRCGS database, since the list contained repeated technical contacts. The response rate was very low, mainly because the contact list found on the website was outdated, so we received many responses of “address not found.” To continue the research, we contracted a survey company to obtain a mailing list referring to the food industry, which carried out the survey and updated the contacts of as many companies as possible contained in the two extracted databases (BRCGS and FSSC 22000). In December 2022, the same questionnaire was sent to 122 technical managers in the production and quality area, now with the contacts updated by the research service provider.

All told, 50 complete responses to the surveys were received. From the standpoint of question standardization, self‐administered questionnaires reduce this source of variability. The most cited disadvantage of mail surveys is the low response rate (Gunther, 2003). On the other hand, (Kronsnick, 1999) cited a research that suggests that low response rates do not necessarily mean a low degree of representation.

### 2.3 Ethical considerations

Before being sent to prospective respondents, the questionnaire was submitted to the Ethics and Research Committee of the Federal University of the State of Rio de Janeiro and was approved under CAAE protocol number 58774322.0.0000.5285.

According to (Conselho Nacional de Saúde, 2012)from the National Health Council (CNS), which deals with research and tests involving human beings, the ethics of research implies respect for the participants’ dignity and autonomy, recognizing their vulnerability, ensuring their willingness to contribute and freedom to withdraw from the survey at any time. Since it was an online questionnaire about the perceptions of those responsible for implementing food safety standards in the company, the participants could choose not to respond.

In addition to the risk factors already mentioned and following (Carta Circular No 1, Ministério da Saúde, 2021) from the National Ethics and Research Commission of the Ministry of Health, the research risks in a virtual environment were considered. Such measures aim to preserve the protection, security, and rights of research participants in vulnerable situations. The survey was also under the National Health Council (CNS) (Conselho Nacional de Saúde, 2016), whereby the use of a free and informed consent form was waived, for the following reasons (i) it was an analytical study, using databases open to public consultation on the certification standards websites; (ii) all data were analyzed anonymously, without nominal identification of the participants and companies consulted; (iii) the results of the study were presented in aggregated form, not allowing the individual identification of participants and companies; and (iv) it was a non‐interventional study (without nutritional and clinical interventions) and without changes/influences on the routine/treatment of the research participants, and consequently without adding risks or harm to their well‐being. All data that were considered sensitive were protected and the respondents were informed that only general data would be used for academic purposes.

### 2.4 Statistics and data analysis

Data analysis was carried out using CA, a statistical technique from the field of multivariate analysis that allows the identification of key dimensions inherent to evaluations made by respondents regarding objects and then positioning such objects on a perceptual map. According to (Hair et al., 2009) as a general rule for creating a perceptual map, a minimum of three attributes and three objects are required, which in this study were represented by the critical factors and the companies evaluated, respectively.

The objective of the CA was to identify the most relevant variables among the critical success factors, to answer the research question. Respondents were asked to classify the variables according to their level of importance.

The CA was performed with the RStudio software (packages base, ca, datasets, factoextra, FactoMineR, ggplot2, ggpubr, graphics, grDiveces, methods, stats, and utils). The analysis was based on similarity data, since the objective of modeling in the present study was to provide a clearer comparison among objects (companies), based on their attributes.

## 3 RESULTS AND DISCUSSION

### 3.1 First section of the questionnaire

Few studies use practical applicability regarding the topic of food defense. Among these is a case study in the dairy industry in Rio de Janeiro (R. Silva et al., 2020), where the objective was to verify the agreement of the respondents with the items covered in a food defense program, carried out by distributing the questionnaire via email and descriptive statistical evaluation of the responses. The results of that work suggested that although a FDP had not been formally implemented, there was concern among the dairy industry regarding this issue, indicating that its formal application could be facilitated.

Also in Brazil, another case study was carried out in a small company that sells meat seasonings located in São Paulo as part of a supply chain supplying export companies that must comply with the legislation in force in the countries of destination of its customers, such as the implementation of food defense.

For this purpose, the FDP Builder Software program was used available on the FDA website that aims to identify vulnerabilities and develop FDPs (Figueira, 2018). It is laborious and time‐consuming, requiring the completion of several steps customized to the company's reality, but it was possible to identify vulnerable points, and based on them develop an action plan for the factory to become safe in terms of intentional contamination actions.

Assessment work on the implementation of food defense requirements was carried out in Lisbon in two 1acking companies (Praia & Henriques, 2021), through a first‐party audit based on a checklist with 116 closed‐response questions, where it was possible to verify the degree of implementation of food defense requirements through the main vulnerabilities detected, which were related to the lack of a FDP, failure to identify critical areas in the company, ineffectiveness of alert systems and lack of knowledge of food defense by employees.

Due to the scarcity of practical references for a food defense implementation in food processing companies, this study was developed with data from firms from different food segments in Brazil, to consolidate information from those responsible for the food safety plans that are certified according to the requirements of food defense. Our overall aim was to extract information to facilitate the implementation or adjustment of these requirements in the food industry.

The survey obtained valid responses from 50 anonymous companies using the codes empr.1 to empr.50. The answers extracted from the first part of the form allowed us to characterize the profile of the responding companies, where 82% had more than 10 years in the market, 14% between 5 and 10 years, and the remaining 4% less than 5 years. Regarding the size classification of companies, 48% were large, with more than 500 employees, 40% were medium, with 100–499 employees and 12% were small, with fewer than 99 employees. Regarding the geographic location of the companies, most are located in the Southeast, South, and Midwest regions of Brazil, with 36%, 34%, and 20%, respectively. As for the industrial segments, 38% were slaughterhouses/meat packing companies, 14% were juice production firms, 4% were involved in rice, coffee, tea, and cereal processing, and the remainder were in miscellaneous other segments.

Regarding exports, of the 50 Brazilian companies responding, only 3 do not export, and the rest of the companies export to more than one destination, namely South America with 70% as the destination for products manufactured in Brazil, followed by Asia with 64%, Europe with 54%, North America with 52%, Central America with 40%, and Africa with 22%.

At the end of the first section of the form, the respondents were asked whether their company had a food industry certification system, and only 1 of the 50 companies did not (a small company and one of those that do not export). Regarding the types of certification, with the possibility of multiples, 76% of respondents had BRC certification, followed by FSSC 22000 with 20%, ISO 22000 representing 10%, HACCP with 6%, ISO 9001 with 4%, and others spread across other important types of certification in the food sector, such as SGS, IFS, PAS 96 and GMP.

### 3.2 Second section of the questionnaire

The second section of the questionnaire elicited responses from 50 food company representatives, who evaluated 30 food defense requirements regarding the level of relevance of the attribute as a critical success factor in protecting food companies from intentional contamination attacks (Table 1).

Calculation of descriptive statistics was the initial step of data analysis, to summarize and understand the data. From Table 2, with the average scores, 29 of the 30 attributes were considered relevant for companies, with the exception being attribute 14.

**TABLE 2:** Average scores per attribute considering the evaluations of the 50 responding company representatives with responses graded according to the scale: “1—Not at all relevant,” “2—Slightly relevant,” “3—Indifferent,” “4—Relevant,” “5—Totally relevant.”

Attribute	Requirements	Average
3	Identification and registration of visitors, and monitoring them throughout the visit	4.82
10	Qualification of suppliers	4.82
29	Employee awareness of food safety and security	4.82
6	Control of access to key stock materials	4.8
5	Control of hazardous materials	4.78
15	Restricted access and monitoring of third‐party access	4.78
30	CCTV monitoring/recording of vulnerable areas	4.76
23	Controlled access to the utilities area (ventilation, air conditioning, water storage, steam system, electrical system, etc.)	4.74
19	Restricted and controlled access of employees to relevant areas	4.7
2	Finished product storage in a specific location	4.66
13	Physicochemical and microbiological control of raw materials	4.66
1	The existence of perimeter fencing throughout the factory	4.64
17	Finished product (retail) packaging with effective seals	4.64
7	Limitations on access to network services	4.6
27	Access cards and keys collected during the dismissal process	4.58
22	CCTV monitoring/recording of perimeter vulnerabilities	4.52
18	Control of packaging labels	4.48
4	Investigation of missed deliveries	4.46
26	Computer accounts closed or suspended during the dismissal process	4.46
8	System for tracking transport vehicles	4.38
9	Monitoring vehicle access points	4.34
21	Restriction on the use of cameras and other portable electronic devices	4.34
16	Routine cyber training (security principles)	4.28
20	Uniforms differentiated by sector	4.24
28	Differentiated recruitment for sensitive functions and/or critical roles (concerning risks)	4.2
25	Access control (entrances and exits) by CHIP and PIN	4.18
12	Providing employees with a list of emergency contacts	4.14
24	Parking of vehicles of employees and visitors outside the industrial sector	4.06
11	The existence of a perimeter alarm system	4.04
14	Proof of identity with criminal record search before hiring new employees	3.66

*Source*: Authors.

This is a broad view, but the attributes are not perceived in the same way by all companies and contribute to explaining data variability.

Table 3 shows the dimensions considered in the solution and their respective eigenvalues. The eigenvalues represent the relative contribution of each dimension in explaining the variance of the categories. The number of dimensions generated is equal to the smallest number between columns (50 companies) and rows (30 food defense requirements) minus 1 company. Therefore, 29 dimensions were generated. They are presented in descending order of eigenvalues, with the first dimension carrying the greatest explanatory power, which decreases until reaching the lowest contribution of individual explanation in dimension 29. Table 1 shows that the total explanatory power, considering all five dimensions, was 60.04%. Each dimension added to the solution increased the explained variance of the solution. However, the complexity of interpreting the results also increased. Therefore, there needs to be a balance between the explanation of the solution and the number of dimensions added (Hair et al., 2009).

**TABLE 3:** Eigenvalues of the five most representative dimensions in the relative contribution of each dimension to explain the variance of the categories, where the total amount of explanation considering five dimensions was 60.04%.

Dimension	Eigenvalue	Variance.percent	Cumulative.variance.percent
Dim.1	3.402394e ‐03	19.00515444	19.00515
Dim.2	2.579319e ‐03	14.40760568	33.41276
Dim.3	1.752825e ‐03	9.79095916	43.20372
Dim.4	1.567457e ‐03	8.75553014	51.95925
Dim.5	1.447341e ‐03	8.08458003	60.04383

*Source*: RStudio Output.

**FIGURE 1:**
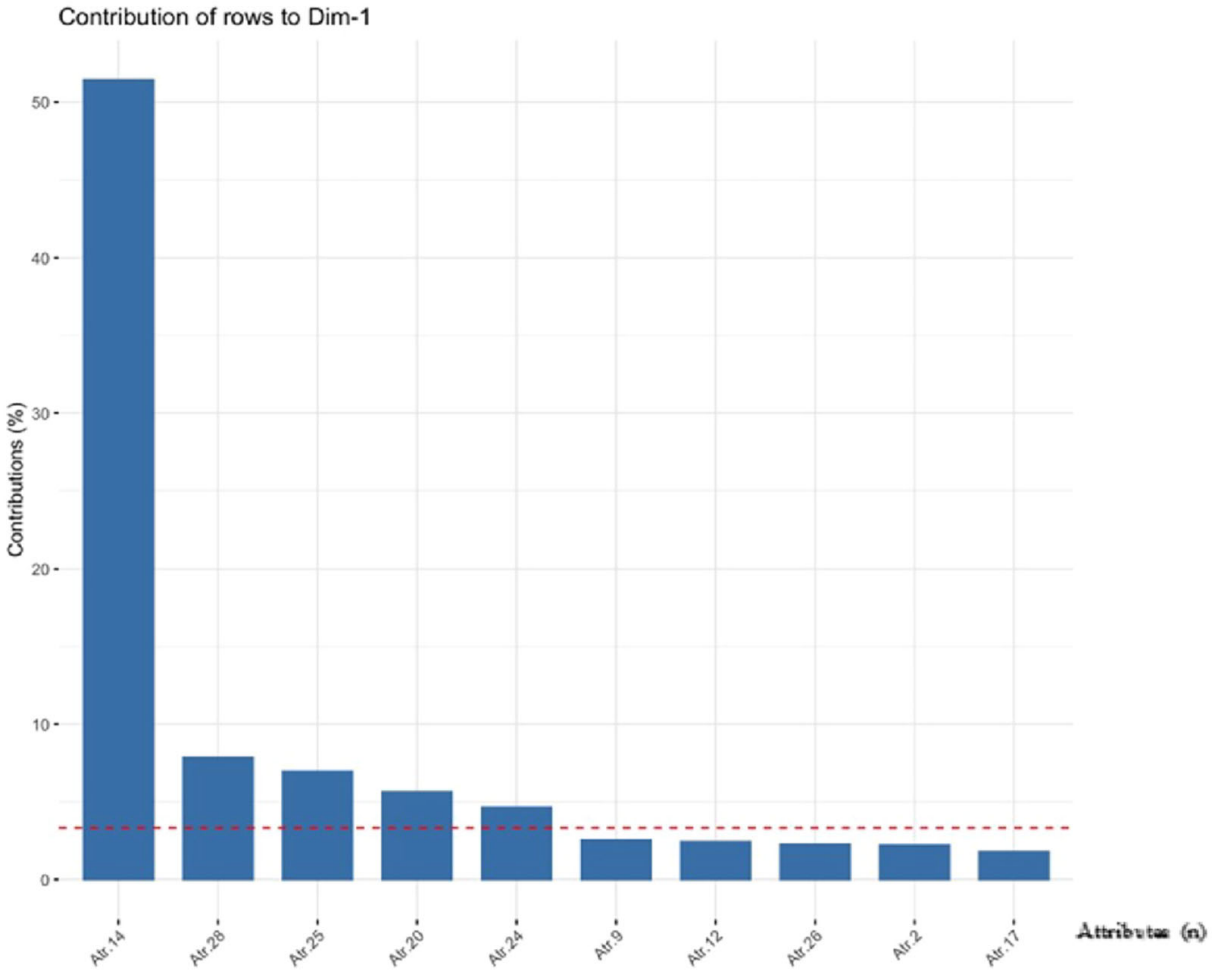
Contribution of attributes to dimension 1 in descending order to allow a visual analysis that most contributes to the model. *Source*: RStudio Output.

**FIGURE 2:**
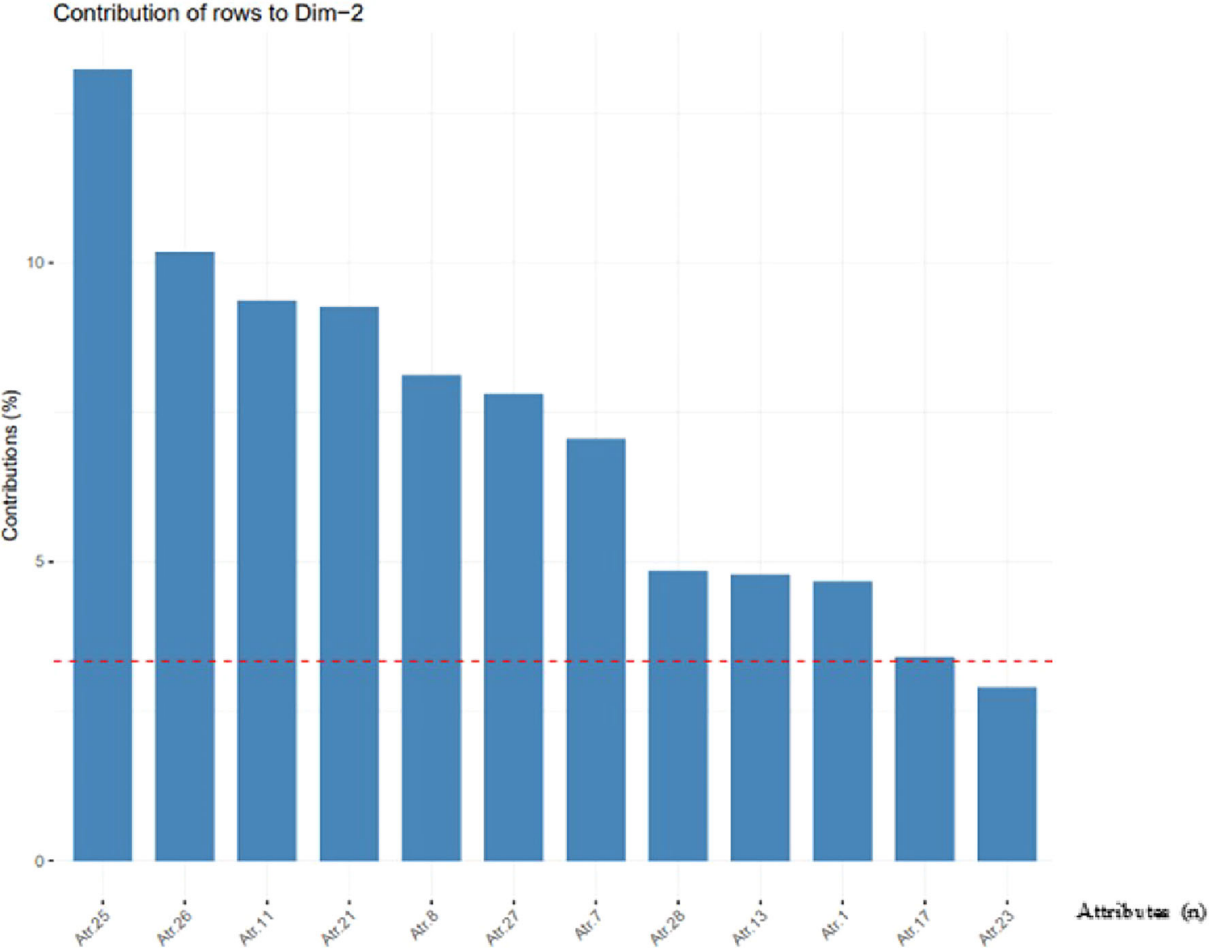
Contribution of attributes to dimension 2 in descending order to allow a visual analysis that most contributes to the model. *Source*: RStudio Output.

To distinguish the attributes that contribute most to the explanation, Figures 1 and 2 were designed to demonstrate the contribution of each attribute in dimensions 1 and 2, respectively. The dashed red line in the figures indicates the expected average value if the contributions of all attributes were uniform.

**FIGURE 3:**
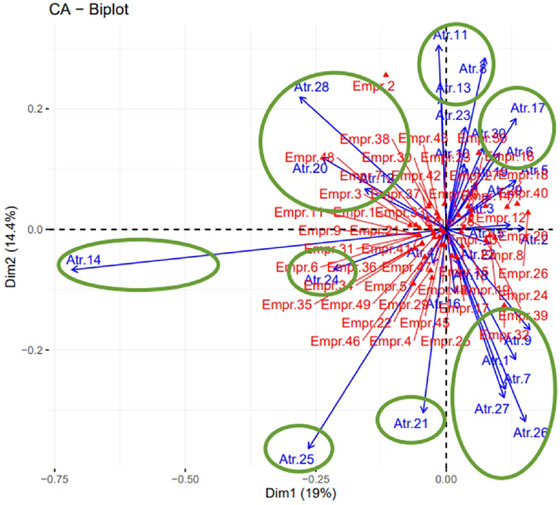
Biplot perceptual map of contribution—two‐dimensional map represented by two axes, displaying the relationship between attributes and companies, and highlighting some attributes for analysis. *Source*: RStudio Output.

From Figures 1 and 2, the analyses were directed concerning the attributes that contributed most to the model. To facilitate the identification of these relationships, we created a contribution biplot perceptual map (Greenacre, 2013) (Figure 3). The perceptual map is a visual representation of the respondents' perceptions regarding the relevance of attributes for food defense implementation. The perceptual map used in this study is two‐dimensional and is represented by two axes, showing the relationship between attributes and companies. In this display, points that contribute very little to the solution are close to the center of the biplot and are relatively unimportant for interpretation (Greenacre, 2013).

Certain attributes were similarly perceived by the respondents, Figure 3 illustrates through ellipses, that four groups were formed and four more highlights for isolated attributes, but with a strong contribution to explaining the CA model.

The first grouping involves Attribute 1 (Existence of perimeter fencing throughout the factory), Attribute 7 (Limitations on access to network services), Attribute 9 (Monitoring of vehicle access points), Attribute 26 (Computer accounts closed or suspended during the dismissal process), and Attribute 27 (Access cards and keys collected during the dismissal process), which are close together and point in the same direction. Similarly, other associations can be made from Figure 3, such as a group of attributes: Attribute 12 (Providing employees with a list of emergency contacts), Attribute20 (Uniforms differentiated by sector), and Attribute 28 (Differentiated recruitment for sensitive functions and/or critical roles (concerning risks), another group formed by Attribute 8 (System for vehicle tracking), Attribute 11 (Existence of a perimeter alarm system, and Attribute13 (Physicochemical and microbiological control of raw materials), and another formed by Attribute 6 (Control of access to key stock materials) and Attribute 17 (Finished product packaging (retail) with effective seals).

Although PAS 96:2017 (The British Standards Institution, 2017) was the reference used to prepare the questionnaire with the requirements and it classifies the requirements into three groups (controlling access, detecting violations, and ensuring the safety of personnel), we used these requirements without grouping, and applied the statistical tool, CA, based on the responses of the respondents. This allowed us to characterize four groups with characteristics similar to those proposed by PAS 96:2017, ratifying the proposal to protect and defend foods and beverages from deliberate attacks.

In Figure 3, the angle between the arrows and the proximity between the points are indicators of association (Hair et al., 2009). Therefore, companies that are on the right side of Figure 3 consider attributes that are also on the right to be more relevant, with the same also about those on the left side. For example, Attribute 24 (Parking of vehicles of employees and visitors outside the industrial sector) is an attribute positioned angularly close for Emp6, Emp.36, and Emp.41. These companies are in the animal slaughter and meat packing segment, and export to North America, Europe, Asia, and Africa. They are also BRC certified and conduct an invasion simulation with an internal agent (someone from within the company).

Attribute 25 (Access control (entrances and exits) by CHIP and PIN), according to Figure 2, has the greatest contribution of attributes to dimension 2, but due to the distance observed in Figure 3, this is not valued in the same way by all companies. Of the 50 companies, 12 evaluated it with a score of 2 (not very relevant) or a score of 3 (indifferent).

Likewise, Attribute 21 (Restriction on the use of cameras and other portable electronic devices) is isolated and has a significant difference when analyzing Figure 3, as only 48% of companies evaluated this attribute with a score of 5 (totally relevant).

About the Attribute 14 (Proof of identity with criminal record search before hiring new employees), it was considered the least relevant among all attributes, since it has the greatest distance from the companies analyzed (Figure 3), being the only one to present a mean below 4 (3.66) and obtaining mode and median equal to 4. This statement can be made with confidence because according to Figure 1, it is the attribute with the greatest contribution in dimension 1. However, some companies classified it as “Totally Relevant,” as was the case of Empr.3 (Slaughter of animals and production of meat and meat products), Empr.6 (Slaughter of animals and production of meat and meat products), and Empr.9 (Production of rice, coffee, tea and cereals).

Further regarding companies, Empr.2 evaluated the relevance of the attributes with the lowest scores, despite stating that the company has the maximum level of food safety culture. In Figure 3, it stands apart from the other companies. This company operates in the production of juices, soft drinks, and beer, is medium‐sized (100–499 employees), is located in the Southeast region, and exports to other countries in South America, North America, Central America, and Europe.

### 3.3 Third section of the questionnaire

Finally, the third part refers to the FDP in relation to the company the respondent represents: regarding the existence of a plan, carrying out the effectiveness testing, and the food safety culture. The results can be seen in Table 4.

**TABLE 4:** Answers responses (%) from the 50 respondents referring to the third section of the questionnaire.

Questions asked	Answers	
	Yes	No
Does the company have a Food Defense Plan?	98%	2%
Are there tests carried out annually of the warning system for potential danger from acts of sabotage, vandalism, or terrorism?	76%	24%
How are these tests carried out?	% Answers	
Simulation with an internal agent (someone from within the company)	68%	
Invasion simulation with an external agent (hiring a third party)	16%	
Simulation with internal and external agent	10%	
Inspection of established control measures	3%	
Internal audit	3%	
In your opinion, what is the company's level of food safety culture?	% Answers	
Minimum	0%	
Neutral	32%	
Maximum	68%	

*Source*: Authors.

Regarding the level of food safety culture, it is important to highlight that we only asked one question about the respondent's opinion regarding the company in which he/she works, to compare with the results of the work as a whole. However, there is the possibility of measuring the level of maturity of the food safety culture through phases and scales referenced in the GFSI Food Safety Culture Maturity Model (GFSI, 2018).

## 4 POLICY IMPLICATIONS

The notion of food defense emerged in the USA, after the terrorist attacks of September 11, 2001, reflecting the idea that the food sector constitutes a critical infrastructure for national security and that the food system must be defended against intentional acts, motivated by political or ideological reasons, which could cause large‐scale damage to public health or the economy (EUA, 2011, 2015). The government's response to these—and previous—food safety problems has been to introduce new food safety legislation or issue improved guidelines to food handling businesses.

Furthermore, food defense can be characterized as a public security problem, as it produces a specific type of discourse that articulates a given issue as an existential threat—that is, as an extreme danger that calls into question the existence of a socially valued object and—a securitizing actor, usually speaking from a position of power, attempts to convince a relevant audience of the need for urgent and exceptional measures to contain the development of the threat (Buzan et al., 1998), that is, the discourse on the bioterrorist threat, constructed at the strategic level of the decisions of American political elites and supported by the acceptance of the scientific community, media, and public opinion, which justified and legitimized the substantive measures that began to be operationalized in food defense practices in the food industries, explaining how the food sector has been securitized.

Awareness of the need to address the food defense concept is gaining pace in the food and beverage industries in Brazil, especially those working with exports. The international market imposes several standards, critical requirements, and protection measures, and Brazilian industries, in order not to lose market share or even to expand their export capacity, must have well‐implemented defense plans to ensure the protection of food against intentional and malicious activity, sabotage, bioterrorism and other points of vulnerability for organizations, forcing Brazilian industries to quickly comply with international legislation. In addition to this driving factor, customers are more informed and therefore more demanding; there is a growing concern among companies regarding customer satisfaction and product differentiation with greater added value, thus the quality attribute becomes a major differentiator of choice for the customer, in addition to the certainty that companies need continuous improvement to survive this reality (da Silva & de Melo, 2017).

As with most food products, US law requires advance notification of foods imported or offered for import into the country. The US has also established a Foreign Supplier Verification Program for importers of human and animal foods, based on a program for the expedited review and import of foods from importers who can maintain a high level of control over safety. of their supply chains (the Qualified Importer Voluntary Program). Both programs came into force in 2018. Food importers must have at least a HACCP program. Furthermore, food products must comply with US labeling standards and separate standards for organic products (CNI, 2018).

Since the FSMA was passed in January 2011 in the USA, more organizations are following food safety regulations. Manufacturers have become more aware of the need to comply with food safety protocols, working diligently to improve safety, quality, accuracy, transparency, and compliance. This was a historic milestone that shifted the focus of food safety from reactive to preventative, where organizations need to prove that they are proactive in ensuring that the food they receive is not harmful to health and is of the nature, substance, and quality required by the purchaser, ensuring transparency and traceability for our collective food supply (Damaren, 2024). The FMSA was created to protect, mitigate, prevent, detect, and respond to intentional contamination in the food supply chain. This law grants the FDA the legislative power to regulate any foreign supplier, entity, or person involved in the preparation of foods in the United States. In May 2016, the FDA released the FSMA final rule that seeks to prevent intentional adulteration of acts intended to cause large‐scale harm to public health, including targeted acts of terrorism to the food supply. Such acts, although unlikely, could cause illness, death, or disruption of the food supply. In this rule, economic adulteration is addressed in the final rules on preventive controls for foods of human and animal origin (FDA, 2016).

In Europe, the most significant food safety standards are the BRC and the IFS. According to these standards, food defense requirements are mandatory and include the implementation of hazard analysis, the assessment of related risks, and the identification of critical areas (British Retailer Consortium, 2015).

In 2018, the ISO 22000 standard (FSMS) published the requirements for a food defense management system. It maps out what an organization needs to do to demonstrate its ability to control food safety risks to ensure food safety. It can be used by any organization, regardless of its size or position in the food chain, from primary production to the consumer (International Organization for Standardization, 2018).

If a food company has partnership agreements or exports products to certain countries, it is obliged to apply the food defense requirements defined by food safety standards and the legislation of the countries with which the company has commercial relations. Food defense is a concern in almost all business areas of the food industry, where intentional contamination and food fraud can pose a series of threats to consumers and public health, in addition to harming the company's business (Bogadi et al., 2016).

Brazil must remain up to date in knowledge and technology to meet the constant changes and demands of consumer markets. This commits Brazil to being prepared to adopt current standards and legislation relevant to food quality and safety (Machado, 2012).

Loader and Hobbs (1999) studied the issue of firm responses to changes in food safety legislation, concluding that companies will respond in different ways depending on their corporate strategies and objectives. However, it is suggested that all companies answer quickly to food safety concerns and that legislation often requires them to do so because they have a particularly direct influence on product marketing and are the source of increasing consumer concerns.

In the area of knowledge construction, although the topics to be addressed must respond to problems relevant to society, the focus becomes the market (demand pull) and technological policy. In this sense, the main influencing element is business demands. Companies have the knowledge and skills to judge what type of science is necessary. Thus, in this paradigm, science is still linear, but the focus of scientific development is influenced by the prioritization made by the productive sector (Velho, 2011).

In summary, what has been observed over the last few years is that food defense practices have started to be disseminated more frequently and in a bureaucratic manner. The rules and regulations of the food sector promote a more active and autonomous involvement of the business sector itself, rather than political speeches, declarations, and official directives, of an emergency nature, which marked the emergence of this concept. It is this more technical and specialized approach, with risk reduction models and tools against intentional adulteration, that has impacted concerns about the protection of the food chain in the government sphere, in the food industry, and in academic debate not only in the USA but also in other regions of the world where the discussion on food defense becomes increasingly necessary and frequent (Andrade et al., 2021; Bogadi et al., 2016; Dalziel, 2009; Fredrickson, 2014; Jurica et al., 2019; Manning & Soon, 2014; Severino & Almeida, 2016).

## 5 CONCLUSIONS

Food defense is a topic that has been little studied in Brazil, and there is scarce information in the public domain on the subject. At the same time, there is an inherent knowledge gap within the industry regarding which food defense strategies need to be addressed, and there is a growing need for food companies to develop and adopt FDPs to ensure market entry and continuity, mainly through certification by outside parties of their management systems (Andrade et al., 2021).

Through profile mapping of the 50 companies, we observed that 47 of the companies analyzed exported their products and had specific food industry certifications. Of the three companies that do not export, only one does not have specific certification. Regarding the types of certifications (highlighting that a company can have more than one type), 76% have BRC certification, followed by FSSC 22000 representing 20%. Both BRC and FSSC22000 are standards recognized by the GFSI, which contemplates a FDP implementation, confirming the requirement for a certified food safety plan for companies that export foods. This statement is supported by the question asked in the last block of the questionnaire, where respondents answered whether the company had a FDP implemented. Again, only the company that does not export and does not have certification responded that it does not. It is imperative for companies seeking to expand their market reach to prioritize the adoption of such certifications and the implementation of robust FDPs. Another important aspect is that of the companies that stated they have a FDP implemented, only 77.5% said they conduct invasion simulation testing of the warning system annually to detect the potential danger of acts of sabotage, vandalism, or terrorism, using an internal or external agent to determine whether the FDP implemented is effective. We urge these companies to consistently test and refine their food defense systems to ensure they are prepared for any potential threats.

Regarding certification type, the BRC standard was the most representative in the sample analyzed. This may have influenced the result because it is the only public consultation database available with email addresses of technical and commercial contacts, by telephone numbers. Although outdated, the database was revised by the research company later.

Returning to the initial objective of this work, which was to contribute to the standardization and prioritization of the requirements necessary to implement food defense in a company based on the analysis of the different standards and the expertise of certified companies, we observed that the attributes and requirements for the implementation of food defense are valued by different ways and companies.

The results discussed in the previous section allowed the formation of four distinct groups, but with intrinsic similarities in the formation of each group, thus allowing these characteristics to create group titles for better understanding by the reader, as shown in Table 5.

**TABLE 5:** Titles of groups of attributes demonstrating proximity with intrinsic similarities are highlighted in the perceptual map.

Attribute	Title
	Access restrictions
1	The existence of perimeter fencing throughout the factory
7	Limitations on access to network services
9	Monitoring vehicle access points
26	Computer accounts closed or suspended during the dismissal process
27	Access cards and keys collected during the dismissal process
	Human resource management
12	Providing employees with a list of emergency contacts
20	Uniforms differentiated by sector
28	Differentiated recruitment for sensitive functions and/or critical roles (concerning risks)
	Controls
11	The existence of a perimeter alarm system
13	Physicochemical and microbiological control of raw materials
8	System for tracking of transport vehicles
	Materials and packaging
6	Control of access to key stock materials
17	Finished product (retail) packaging with effective seals

*Source*: Authors.

This result comes from the subtlety that the CA method provides because even unconsciously, some attributes are perceived similarly by the respondents, enabling the formation of groups. This means that when implementing food defense requirements in companies, joint efforts can be emphasized and concentrated on requirements belonging to the themes that include “access restrictions,” “human resources management,” “controls,” and “materials and packaging.” Companies should prioritize these areas to build a comprehensive and effective food defense strategy, especially those with limited resources.

Likewise, from the results, we can infer that there is no need, priority, or urgency to implement Attribute 14 (proof of identity with criminal record search before hiring new employees), as mentioned in the previous analysis.

For similar analysis, starting with companies in relation to attributes, that is, trying to characterize groups of companies with similar affinities in relation to food defense requirements, we suggest future research with more detailed characterization of the companies or the respondent, to identify the existence or not of similarities between groups of companies, which can also support the formation of new affinity groups about attributes and thus facilitate the implementation or adaptation of food defense requirements in the food industry in general. We encourage researchers to investigate these patterns before refining and enhancing food defense strategies.

Based on the identification and mapping of the critical success factors for implementing food defense, our results can contribute to choices on implementing food defense or the need to adjust the requirements for maintaining a FDP. This can serve as a guide for food companies to develop effective strategies to face the threats of intentional contamination and protect consumer health, thus contributing to food safety for consumers and economic growth through new entrants in the food industry in the export and import chain.

Finally, it is important to highlight the results from the last question of the questionnaire, on the level of food safety culture, where 68% of respondents said that their company has the maximum level of culture, and 32% responded that they had a neutral level, although 98% of the respondents said their company had a FDP in place. According to the GFSI, food safety culture is defined as: “shared values, beliefs, and norms that affect mindset and behavior toward food safety in, across and throughout an organization” (GFSI, 2018), In other words, food safety culture is what ensures the continued maintenance of an effective FDP that is regularly updated and guarantees the supply of safe food to the public. Therefore, we suggest the future of how to measure and improve food safety culture in organizations.

Given the critical findings in our study, we strongly encourage companies to initiate the implementation of a FDP, leveraging the identified success factors. By actively engaging in these practices, companies can not only enhance their food defense mechanisms but also ensure that their operations align with global standards, thus securing consumer trust and expanding market opportunities. Furthermore, applying our model in companies across different countries will validate its broader applicability and foster international collaboration in food safety initiatives.

## Supporting information



Supporting Information

## References

[risa70025-bib-0001] Andrade, E. L. I. d. M. , de Oliveira, G. C. , & Silva, O. F. (2021). Food defense—Do conceito às atuais exigências do mercado internacional. Research, Society and Development, 10(17), e201101724175. 10.33448/rsd-v10i17.24175

[risa70025-bib-0002] Bogadi, N. P. , Banović, M. , & Babić, I. (2016). Food defence system in food industry: Perspective of the EU countries. Journal fur Verbraucherschutz und Lebensmittelsicherheit, 11(3), 217–226. 10.1007/s00003-016-1022-8

[risa70025-bib-0003] British Retailer Consortium . (2015). BRC—Global Standard Food Safety—Issue 7 frequently asked questions. https://www.brcglobalstandards.com/media/63848/brc_global_standard_for_food_safety_issue_7_faqs‐1.pdf

[risa70025-bib-0004] Buchanan, R. L. , & Appel, B. (2010). Combining analysis tools and mathematical modeling to enhance and harmonize food safety and food defense regulatory requirements. International Journal of Food Microbiology, 139(Suppl 1), S48–S56. 10.1016/j.ijfoodmicro.2010.01.015 20149936

[risa70025-bib-0005] Buzan, B. , & Wæver, O. , de Wilde, J. (1998). Security: A new framework of analysis. Lynne Rienner.

[risa70025-bib-0006] Carta Circular No 1, Ministério da Saúde . (2021). https://conselho.saude.gov.br/images/comissoes/conep/documentos/CARTAS/Carta_Circular_01.2021.pdf

[risa70025-bib-0007] CNA . (2021). Panorama do Agro. Sistema CNA /SENAR / Instituto CNA. https://www.cnabrasil.org.br/cna/panorama‐do‐agro

[risa70025-bib-0053] CNI . (2018). Relatório sobre as principais dificuldades e requisitos de acesso aos Estados Unidos que afetam as exportações brasileiras. https://www.apexbrasil.com.br/Content/imagens/5c5ac1dc‐21e3‐4cd4‐b2dc‐a597472d83a1.pdf

[risa70025-bib-0049] Cooper, D. R. , & Schindler, P. S. (2003). Research methods. Boston, MA: Irwin.

[risa70025-bib-0009] Conselho Nacional de Saúde . (2012). Resolução no 466 . https://conselho.saude.gov.br/resolucoes/2012/Reso466.pdf

[risa70025-bib-0010] Conselho Nacional de Saúde . (2016). Resolução no 510 . https://conselho.saude.gov.br/resolucoes/2016/Reso510.pdf

[risa70025-bib-0011] Creswell, J. W. (2007). Projeto de pesquisa: métodos qualitativo, quantitativo e misto (2nd ed.).

[risa70025-bib-0012] Dalziel, G. R. (2009). Food defence incidents 1950–2008: A chronology and analysis of incidents involving the malicious contamination of the food supply chain. (Technical Report No 66). Centre of Excellence for National Security (CENS). http://www.food‐defense.it/1/upload/rsis_food_defence_170209.pdf

[risa70025-bib-0013] Damaren, P. (2024). Focus on food safety and transparency. Food Safety News, 1–4. https://www.foodsafetynews.com/2024/01/focus‐on‐food‐safety‐and‐transparency

[risa70025-bib-0014] EUA . (2011). Sector critical infrastructure protection (Annual Report for the Food and Agriculture Sector) .

[risa70025-bib-0015] EUA . (2015). Report to Congress on the National Agriculture and Food Defense Strategy (NAFDS) Submitted pursuant to Section 108 of the FDA Food Safety Modernization Act (FSMA), Public Law 111–353 .

[risa70025-bib-0016] FDA . (2016). FDA Food Safety Modernization Act .

[risa70025-bib-0017] FDA . (2019). Mitigation strategies to protect food against intentional adulteration: Guidance for industry revised draft guidance (Issue March 2019) . https://www.fda.gov/media/113684/download

[risa70025-bib-0052] Figueira, C. L. (2018). Os conceitos de Defesa dos Alimentos (Food Defense) e Fraude em Alimentos (Food Fraud) aplicados em fábrica de temperos cárneos–um estudo de caso. 2018.81 f (Doctoral dissertation, Dissertação (mestrado)–Faculdade de Zootecnia e Engenharia de Alimentos, Universidade de São Paulo, Pirassununga).

[risa70025-bib-0018] Forsythe, S. J. (2013). Microbiologia da Segurança dos Alimentos (2nd ed.).

[risa70025-bib-0019] Fredrickson, N. R. (2014). Food security: Food defense and biosecurity. In Encyclopedia of agriculture and food systems (Vol. 3). Elsevier Ltd. 10.1016/B978-0-444-52512-3.00036-X

[risa70025-bib-0020] Food Safety System Certification 22000 . (2019). Guidance document: Food fraud mitigation . https://www.fssc22000.com/wp‐content/uploads/19.0528‐Guidance_Food‐Defense_Version‐5.pdf

[risa70025-bib-0021] GFSI . (2018). Cultura de segurança de alimentos . https://mygfsi.com/news‐and‐resources/?type=publications&topic=food‐safety‐culture&lang

[risa70025-bib-0022] GFSI Benchmarking Requirements Version 2020 . (2020). https://mygfsi.com/wp‐content/uploads/2020/02/GFSI‐Benchmarking‐Requirements‐v2020.1‐3.zip

[risa70025-bib-0023] Greenacre, M. (2013). Contribution biplots. Journal of Computational and Graphical Statistics, 22, 107–122.

[risa70025-bib-0055] Guide to developing a food defense plan for food processing plants . (2008). https://www.fsis.usda.gov/sites/default/files/media_file/2020‐07/Guidance_Document_Warehouses.pdf

[risa70025-bib-0024] Gunther, H. (2003). Como elaborar um Questionário (Série: Planejamento de Pesquisa nas Ciências Sociais, N 01) . UNB, Laboratório de Psicologia Ambiental. www.psi‐ambiental.net/pdf/01Questionario.pdf

[risa70025-bib-0025] Hair, J. F. , Black, W. C. , Babin, B. J. , Anderson, R. E. , & Tatham, R. L. (2009). Análise multivariada de dados (6th ed.). Bookman.

[risa70025-bib-0026] International Organization for Standardization . (2018). *Food safety management systems—Requirements for any organization in the food chain* (ISO 22000:2018). https://www.iso.org/standard/65464.html

[risa70025-bib-0027] Jurica, K. , Vrdoljak, J. , & Karačonji, I. B. (2019). Food defence systems as an answer to food terrorism. Arhiv Za Higijenu Rada i Toksikologiju, 70(4), 232–255. 10.2478/aiht-2019-70-3357 32623862

[risa70025-bib-0028] Kronsnick, J. A. (1999). Survey research. In Department of Psychology (Ed.), *Survey research* (pp. 537–567). Ohio State University.

[risa70025-bib-0029] Loader, R. , & Hobbs, J. E. (1999). Strategic responses to food safety legislation. Food Policy, 24(6), 685–706. 10.1016/S0306-9192(99)00073-1

[risa70025-bib-0048] Lorenzen, C. L. , Hendrickson, M. K. , Weaber, R. L. , Clarke, A. D. , Shannon, M. C. , & Savage‐Clarke, K. L. (2009). Food Defense ‐ Protecting the food supply from intentional harm. https://extension.missouri.edu/media/wysiwyg/Extensiondata/Pub/pdf/miscpubs/mp0914.pdf

[risa70025-bib-0030] Machado, S. S. (2012). Gestão da qualidade (pp. 40‐44). Rede E‐Tec Brasil.

[risa70025-bib-0031] Manning, L. , & Soon, J. M. (2014). Developing systems to control food adulteration. Food Policy, 49(P1), 23–32. 10.1016/j.foodpol.2014.06.005

[risa70025-bib-0046] Manning, L. , & Soon, J. M. (2016). Food Safety, Food Fraud, and Food Defense: A Fast Evolving Literature. Journal of Food Science, 81(4), R823–R834. 10.1111/1750-3841.13256 26934423

[risa70025-bib-0032] Money, A. H. , Babin, B. , & Samouel, P. (2005). Fundamentos de métodos de pesquisa em Administração. Bookman.

[risa70025-bib-0033] Mota, J. da S. (2019). Utilização do Google forms na pesquisa acadêmica. Humanidades & Inovação, 6(12), 371–380.

[risa70025-bib-0047] Motarjemi, Y. , & Lelieveld, H. (2014). Food Safety Management ‐ A Pratical Guide for the Food Industry. Academic Press.

[risa70025-bib-0034] Narrod, C. , Dou, X. , Chfadi, T. , & Miller, M. (2021). Participant characteristics and learning outcomes: Lessons from international food safety capacity building. Food Policy, 102(May), 102105. 10.1016/j.foodpol.2021.102105

[risa70025-bib-0035] Office of the Federal Register . (2013). Federal Register (Vol. 78, Issue 247). https://www.govinfo.gov/app/details/FR‐2013‐12‐24/2013‐30373/context

[risa70025-bib-0036] Praia, E. F. , & Henriques, A. R. (2021). Assessing the implementation of food defense requirements in industrial meat‐based food processors. Brazilian Journal of Food Technology, 24, 1–14. 10.1590/1981-6723.20120

[risa70025-bib-0037] Sapsford, R. (2007). Survey research (2nd ed.). SAGE Publications. 10.4135/9780857024664

[risa70025-bib-0054] Severino, P. , & Almeida, D. (2016). Food Defense – Sistemas de gestão contra o terrorismo alimentar. Agrobook.

[risa70025-bib-0050] Siegel, S. , & Castellan Jr, N. J. (2006). Estatística não‐paramétrica para ciências do comportamento. Artmed Editora.

[risa70025-bib-0051] Silva, R. , Batista, A. L. D. , Azeredo, D. R. P. , Esmerino, E. A. , & da Cruz, A. G. (2020). Defesa dos alimentos (food defense): estudo de caso em laticínio no Rio de Janeiro. Alimentos: Ciência, Tecnologia e Meio Ambiente, 1(2), 1–10.

[risa70025-bib-0045] States, U. (2011). Public Law 111‐353 Food Safety Modernization Act, 4/1/2011, 111th Congress, 124 Stat 3885.

[risa70025-bib-0039] da Silva, A. M. , & de Melo, R. M. (2017). Uma abordagem multicritério para a seleção de serviços de consultoria e certificação de Sistemas de Gestão da Qualidade. Gestão & Produção, 25(1), pp. 160–174. 10.1590/0104-530x2753-16

[risa70025-bib-0040] The British Standards Institution . (2017). Guide to protecting and defending food and drink from deliberate attack. (BSI Standards Limited PAS 96:2017). Department for Environment Food & Rural Affairs. https://www.food.gov.uk/sites/default/files/media/document/pas962017_0.pdf

[risa70025-bib-0041] U.S. Department of Agriculture . (2023). Food defense . https://www.fsis.usda.gov/food‐safety/food‐defense‐and‐emergency‐response/food‐defense

[risa70025-bib-0042] Velho, L. (2011). Conceitos de ciência e a política científica, tecnológica e de inovação. Sociologias, 13, 128–153. 10.1590/S1517-45222011000100006

[risa70025-bib-0043] Weber, C. (2024). Global Food Safety Initiative (GFSI): Ensuring global food safety standards. Registrar Corp.

[risa70025-bib-0044] World Health Organization . (2007). The world health report 2007: A safer future: Global public health security in the 21st century . https://apps.who.int/iris/handle/10665/43713

