# Supplementary material for: Critical monitoring and control factors for achieving food defense criteria
Source: Risk Anal. 2025 Apr 4;45(8):2427–41. doi: 10.1111/risa.70025 (PMC12411119; doi:10.1111/risa.70025)
Supplement: Supplementary file 1 — Supporting Information [file RISA-45-2427-s001.docx]

**SUPPLEMENTAL MATERIAL - QUESTIONNAIRE STRUCTURE**

| **Food defense: critical success factors for implementation in Brazilian companies** | | | | | | |
| --- | --- | --- | --- | --- | --- | --- |
| **As a member of the quality or production area team, we invite you to participate in the research project "Food defense: critical success factors for implementation in Brazilian companies"…...** | | | | | | |
| **Identifying the priority criteria for implementing food defense in Brazilian companies is the general objective of this study, which uses this questionnaire as a data collection instrument, designed to maintain the anonymity of the participants and the company they represent through generic data.** | | | | | | |
| **Participation in the study involves filling out a questionnaire divided into 3 parts:** | | | | | | |
| **The first part aims to collect information about the company and certifications;** | | | | | | |
| **The second part covers food defense requirements, the purpose of which is to evaluate your perception regarding the relevance of each requirement, in order to guarantee the protection of the food producing company from an intentional contamination attack;** | | | | | | |
| **The third part refers to the food defense plan and the culture of food security.** | | | | | | |
| **By answering the questionnaire, you agree to participate in the study. You can withdraw from the study at any time.** | | | | | | |
| **We appreciate your attention!** | | | | | | |
| **Email for research-related questions:** | | | | | | |
| **COMPANY CHARACTERIZATION** | | | | | | |
| The company is: ( ) ≤ 5 years old ( ) between 5 and 10 years old ( )≥ 10 years old | | | | | | |
| The company is classified as: | | | | | | |
| ( ) Small (up to 99 employees) ( ) Medium (100 to 499 employees) ( ) Large (over 500 employees) | | | | | | |
| Geographic location of the company: | | | | | | |
| ( ) Southeast Region of Brazil ( ) Southern Region of Brazil ( ) Northeast Region of Brazil ( ) | | | | | | |
| Midwest Region of Brazil ( ) Northern Region of Brazil | | | | | | |
| 4.Industry Segment: | | | | | | |
| ( ) Sugar manufacturing and refining; | | | | | | |
| ( ) Milling wheat, manufacturing pasta and bakery products; | | | | | | |
| ( ) Production of oils and fats (grain pressing, production of bran, vegetable oils and animal fat); | | | | | | |
| ( ) Slaughter of animals and production of meat and meat products; | | | | | | |
| ( ) Processing of milk and manufacturing of dairy products; | | | | | | |
| ( ) Manufacture of animal feed and food; | | | | | | |
| ( ) Juice production; | | | | | | |
| ( ) Production of soft drinks and/or beers; | | | | | | |
| ( ) Processing of rice, coffee, tea and cereals; | | | | | | |
| ( ) Others, which: ________________________________________________________________________ | | | | | | |
| Does the company export its products to another country? ( ) Yes No | | | | | | |
| If so, where to. Mark all that fit | | | | | | |
| ( ) South America ( ) North America ( ) Central America ( ) Europe ( ) Africa ( ) Asia ( ) Oceania | | | | | | |
| Does the company have a HACCP system? ( )Yes ( )No | | | | | | |
| Does the company have any type of certification? ( )Yes ( )No | | | | | | |
| If so, which ones? | | | | | | |
| ( ) ISO 22000 | | | | | | |
| ( ) FSSC 22000 | | | | | | |
| ( ) PAS 96 | | | | | | |
| ( ) BRC | | | | | | |
| ( ) IFS Food | | | | | | |
| ( ) SQF | | | | | | |
| ( ) Others. Which: ______________________________ | | | | | | |
|  | | | | | | |
| In this section is presented the food defense requirements. Indicate your perception regarding the level of relevance of each of them, as critical success factors in protecting any food company against an intentional contamination attack. | | | | | | |
| Important: In this section, your answers are not in relation to the company for which you work, but in relation to the food industry in general. | | | | | | |
| **Attributes** | **Response scale** | | | | | |
|  | Not relevant | Not very relevant | Indifferent | Very relevant | Totally relevant | Not applicable |
| Controlled access to the utilities area (ventilation, air conditioning, water storage, steam system, and electrical system, etc.) | ( ) | ( ) | ( ) | ( ) | ( ) | ( ) |
| Parking of vehicles of employees and visitors outside the industrial sector | ( ) | ( ) | ( ) | ( ) | ( ) | ( ) |
| Existence of perimeter fencing throughout the factory | ( ) | ( ) | ( ) | ( ) | ( ) | ( ) |
| Existence of a perimeter alarm system | ( ) | ( ) | ( ) | ( ) | ( ) | ( ) |
| CCTV monitoring/recording of perimeter vulnerabilities | ( ) | ( ) | ( ) | ( ) | ( ) | ( ) |
| Monitoring of vehicle access points | ( ) | ( ) | ( ) | ( ) | ( ) | ( ) |
| Investigation of missed deliveries | ( ) | ( ) | ( ) | ( ) | ( ) | ( ) |
| Access control (entrances and exits) by CHIP and PIN | ( ) | ( ) | ( ) | ( ) | ( ) | ( ) |
| Uniforms differentiated by sector | ( ) | ( ) | ( ) | ( ) | ( ) | ( ) |
| Routine cyber training (security principles) | ( ) | ( ) | ( ) | ( ) | ( ) | ( ) |
| Employee awareness of food safety and security | ( ) | ( ) | ( ) | ( ) | ( ) | ( ) |
| Identification and registration of visitors, and monitoring them throughout the visit | ( ) | ( ) | ( ) | ( ) | ( ) | ( ) |
| Physicochemical and microbiological control of the raw materials. | ( ) | ( ) | ( ) | ( ) | ( ) | ( ) |
| CCTV monitoring/recording of vulnerable areas | ( ) | ( ) | ( ) | ( ) | ( ) | ( ) |
| Restriction on the use of cameras and other portable electronic devices | ( ) | ( ) | ( ) | ( ) | ( ) | ( ) |
| Limitations on access to network services | ( ) | ( ) | ( ) | ( ) | ( ) | ( ) |
| Finished product storage in a specific location | ( ) | ( ) | ( ) | ( ) | ( ) | ( ) |
| Control of packaging labels | ( ) | ( ) | ( ) | ( ) | ( ) | ( ) |
| Finished product (retail) packaging with effective seals | ( ) | ( ) | ( ) | ( ) | ( ) | ( ) |
| Control of hazardous materials | ( ) | ( ) | ( ) | ( ) | ( ) | ( ) |
| Control of access to key stock materials | ( ) | ( ) | ( ) | ( ) | ( ) | ( ) |
| System for tracking of transport vehicle | ( ) | ( ) | ( ) | ( ) | ( ) | ( ) |
| Proof of identity with criminal record search before hiring new employees | ( ) | ( ) | ( ) | ( ) | ( ) | ( ) |
| Restricted and controlled access of employee to relevant areas | ( ) | ( ) | ( ) | ( ) | ( ) | ( ) |
| Qualification of suppliers | ( ) | ( ) | ( ) | ( ) | ( ) | ( ) |
| Differentiated recruitment for sensitive functions and/or critical roles (in relation to risks) | ( ) | ( ) | ( ) | ( ) | ( ) | ( ) |
| Restricted access and monitoring of third parties’ access | ( ) | ( ) | ( ) | ( ) | ( ) | ( ) |
| Access cards and keys collected during the dismissal process | ( ) | ( ) | ( ) | ( ) | ( ) | ( ) |
| Computer accounts closed or suspended during the dismissal process | ( ) | ( ) | ( ) | ( ) | ( ) | ( ) |
| Providing employees with a list of emergency contacts | ( ) | ( ) | ( ) | ( ) | ( ) | ( ) |
| Does the company have a food defense plan? ( ) Yes ( )No | | | | | | |
| Are there tests carried out annually of the warning system for potential danger from acts of sabotage, vandalism or terrorism? ( )Yes ( )No | | | | | | |
| If so, how are these tests performed? | | | | | | |
| ( ) simulation with an internal agent (someone from within the company) | | | | | | |
| ( ) invasion simulation with an external agent (hiring a third party) | | | | | | |
| ( ) other way. Please describe: | | | | | | |
| In your opinion, what is the company’s level of food safety culture: | | | | | | |
| ( ) Minimum ( ) neutral ( ) maximum | | | | | | |

Source: Adapted from Ferreira (2018)
